# 1-(2-Bromo­ethyl)-1,4-diazo­niabicyclo­[2.2.2]octane bromide dihydrogen phosphate–phospho­ric acid (1/1)

**DOI:** 10.1107/S1600536810017071

**Published:** 2010-05-15

**Authors:** Jing-Mei Xiao

**Affiliations:** a Ordered Matter Science Research Center, College of Chemistry and Chemical Engineering, Southeast University, Nanjing 211189, People’s Republic of China

## Abstract

In the crystal structure of the title compound, C_8_H_17_BrN_2_^2+^·Br^−^·H_2_PO_4_^−^·H_3_PO_4_, the cations, anions and phospho­ric acid mol­ecules are linked by O—H⋯O, N—H⋯O and O—H⋯Br hydrogen bonds into layers parallel to (101).

## Related literature

For the dielectric properties of *N*-protonated compounds, see: Szafranski & Katrusiak (2008 ▶); Katrusiak & Szafranski (1999 ▶); Chen *et al.* (2008 ▶); Fu *et al.* (2009 ▶); Zhao *et al.* (2008 ▶).
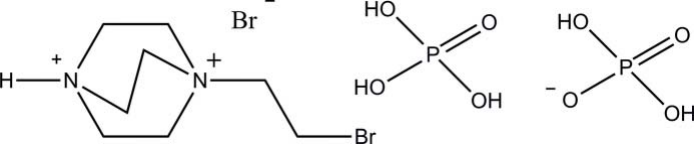


## Experimental

### 

#### Crystal data

C_8_H_17_BrN_2_^2+^·Br^−^·H_2_PO_4_^−^·H_3_PO_4_
*M* *_r_* = 496.02
Monoclinic, 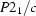

*a* = 12.963 (3) Å
*b* = 7.5959 (15) Å
*c* = 17.721 (4) Å
β = 92.00 (3)°
*V* = 1743.9 (7) Å^3^
*Z* = 4
Mo *K*α radiation
μ = 4.87 mm^−1^
*T* = 293 K
0.3 × 0.25 × 0.2 mm

#### Data collection

Rigaku Mercury2 diffractometer
Absorption correction: multi-scan (*CrystalClear*; Rigaku, 2005 ▶) *T*_min_ = 0.240, *T*_max_ = 0.379
17431 measured reflections
3991 independent reflections
3097 reflections with *I* > 2σ(*I*)
*R*_int_ = 0.072

#### Refinement

*R*[*F* ^2^ > 2σ(*F* ^2^)] = 0.057
*wR*(*F* ^2^) = 0.147
*S* = 1.10
3991 reflections
199 parameters
H-atom parameters constrained
Δρ_max_ = 1.03 e Å^−3^
Δρ_min_ = −1.30 e Å^−3^

### 

Data collection: *CrystalClear* (Rigaku, 2005 ▶); cell refinement: *CrystalClear*; data reduction: *CrystalClear*; program(s) used to solve structure: *SHELXS97* (Sheldrick, 2008 ▶); program(s) used to refine structure: *SHELXL97* (Sheldrick, 2008 ▶); molecular graphics: *SHELXTL* (Sheldrick, 2008 ▶); software used to prepare material for publication: *SHELXL97*.

## Supplementary Material

Crystal structure: contains datablocks I, global. DOI: 10.1107/S1600536810017071/rz2443sup1.cif

Structure factors: contains datablocks I. DOI: 10.1107/S1600536810017071/rz2443Isup2.hkl

Additional supplementary materials:  crystallographic information; 3D view; checkCIF report

## Figures and Tables

**Table 1:** Hydrogen-bond geometry (Å, °)

*D*—H⋯*A*	*D*—H	H⋯*A*	*D*⋯*A*	*D*—H⋯*A*
N1—H1*C*⋯O2	0.91	1.79	2.667 (6)	162
O1—H1*D*⋯O6	0.82	1.71	2.519 (5)	170
O4—H4*C*⋯O3^i^	0.82	1.80	2.560 (5)	155
O5—H5*C*⋯O3^i^	0.82	1.73	2.555 (5)	179
O7—H7*C*⋯O2^ii^	0.82	1.77	2.555 (5)	159
O8—H8*D*⋯Br1^iii^	0.96	2.18	3.100 (4)	160

Symmetry codes: (i) 
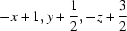
; (ii) 
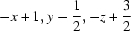
; (iii) 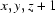.

**Fig. 1.**
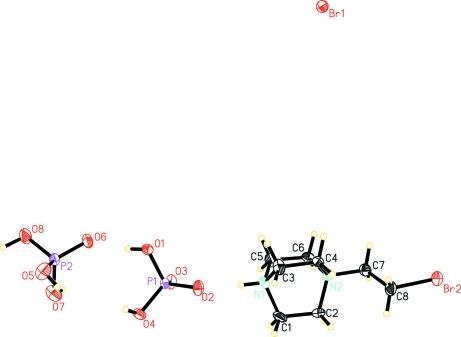
The molecular structure of the title compound, showing the atomic numbering scheme. Displacement ellipsoids are drawn at the 30% probability level.

**Fig. 2.**
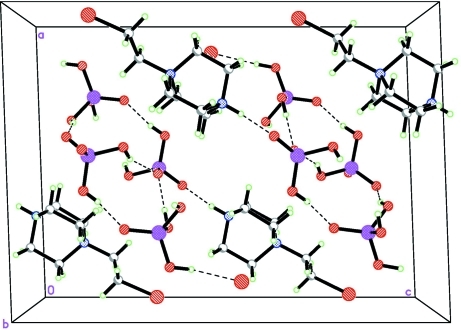
A view of the packing of the title compound, stacking along the b axis. Dashed lines indicate hydrogen bonds.

## supplementary crystallographic information

### Comment

The variable-temperature dielectric response, especially in relatively high frequency range, is very useful for searching phase transitions in which there is a dielectric anomaly at the transition temperature (Szafranski & Katrusiak, 2008; Katrusiak & Szafranski, 1999; Chen *et al.*, 2008; Fu *et al.*, 2009; Zhao *et al.*, 2008). Unluckily, the title compound, whose crystal structure is reported herein, has no dielectric disuniformity from 93 K to 400 K (m.p. = 410-412 K).

The asymmetric unit of the title compound (Fig. 1) consists of a 1-(2-bromoethyl)-1,4-diazabicyclo[2.2.2]octane-1,4-diium dication, a bromide anion, a dihydrogenphosphate anion and a phosphoric acid molecule. In the crystal packing (Fig. 2), intermolecular N—H···O, O—H···O and O—H···Br hydrogen interactions (Table 1) link ions and molecules forming layers parallel to the (101) plane.

### Experimental

To a solution of 1,4-diazabicyclo[2.2.2]octane (5.6 g, 0.05 mol) in chloroform (20 ml) 1,2-dibromoethane (0.048 mol) was added in one portion and the mixture was refluxed for 8 hour. On standing for about 16 hour at room temperature, colourless hygroscopic crystals of 4-diazabicyclo[2.2.2]octan-1-ium bromide were obtained. The crude product was collected and dissolved in 20 ml methanol, then 1 M H_3_PO_4_ was dropped slowly with stirring. The white precipitate formed (yield 80%) was filtered and dissolved in the H_2_O. After a few weeks, colourless hygroscopic block crystals of the title compound were obtained on slow evaporation of the solvent.

### Refinement

All the H atoms were calculated geometrically and refined using a riding model, with N—H = 0.90 Å, C—H = 0.97 Å, O—H = 0.82 Å, and with U_iso_(H) = 1.2U_eq_(C, N) or  1.5U_eq_(O).

### Crystal data

**Table:**

C_8_H_17_BrN_2_^2+^·Br^−^·H_2_PO_4_^−^·H_3_PO_4_	*F*(000) = 992
*M**_r_* = 496.02	*D*_x_ = 1.889 Mg m^−^^3^
Monoclinic, *P*2_1_/*c*	Mo *K*α radiation, λ = 0.71073 Å
Hall symbol: -P 2ybc	Cell parameters from 7671 reflections
*a* = 12.963 (3) Å	θ = 3.4–27.5°
*b* = 7.5959 (15) Å	µ = 4.87 mm^−^^1^
*c* = 17.721 (4) Å	*T* = 293 K
β = 92.00 (3)°	Prism, colourless
*V* = 1743.9 (7) Å^3^	0.3 × 0.25 × 0.2 mm
*Z* = 4	

### Data collection

**Table:**

Rigaku Mercury2 diffractometer	3097 reflections with *I* > 2σ(*I*)
Radiation source: fine-focus sealed tube	*R*_int_ = 0.072
graphite	θ_max_ = 27.5°, θ_min_ = 3.1°
CCD_Profile_fitting scans	*h* = −16→16
Absorption correction: multi-scan (*CrystalClear*; Rigaku, 2005)	*k* = −9→9
*T*_min_ = 0.240, *T*_max_ = 0.379	*l* = −23→22
17431 measured reflections	1814 standard reflections
3991 independent reflections	

### Refinement

**Table:**

Refinement on *F*^2^	Primary atom site location: structure-invariant direct methods
Least-squares matrix: full	Secondary atom site location: difference Fourier map
*R*[*F*^2^ > 2σ(*F*^2^)] = 0.057	Hydrogen site location: inferred from neighbouring sites
*wR*(*F*^2^) = 0.147	H-atom parameters constrained
*S* = 1.10	*w* = 1/[σ^2^(*F*_o_^2^) + (0.0703*P*)^2^ + 1.8668*P*] where *P* = (*F*_o_^2^ + 2*F*_c_^2^)/3
3991 reflections	(Δ/σ)_max_ < 0.001
199 parameters	Δρ_max_ = 1.03 e Å^−^^3^
0 restraints	Δρ_min_ = −1.29 e Å^−^^3^

### Special details

**Table:**

Geometry. All esds (except the esd in the dihedral angle between two l.s. planes) are estimated using the full covariance matrix. The cell esds are taken into account individually in the estimation of esds in distances, angles and torsion angles; correlations between esds in cell parameters are only used when they are defined by crystal symmetry. An approximate (isotropic) treatment of cell esds is used for estimating esds involving l.s. planes.
Refinement. Refinement of *F*^2^ against ALL reflections. The weighted *R*-factor wR and goodness of fit *S* are based on *F*^2^, conventional *R*-factors *R* are based on *F*, with *F* set to zero for negative *F*^2^. The threshold expression of *F*^2^ > σ(*F*^2^) is used only for calculating *R*-factors(gt) etc. and is not relevant to the choice of reflections for refinement. *R*-factors based on *F*^2^ are statistically about twice as large as those based on *F*, and *R*- factors based on ALL data will be even larger.

### Fractional atomic coordinates and isotropic or equivalent isotropic displacement parameters (Å^2^)

**Table:**

	*x*	*y*	*z*	*U*_iso_*/*U*_eq_	
C1	0.8045 (5)	0.7487 (8)	0.5147 (3)	0.0471 (14)	
H1A	0.8221	0.6458	0.5450	0.057*	
H1B	0.8240	0.8527	0.5436	0.057*	
C2	0.8627 (4)	0.7443 (7)	0.4414 (3)	0.0352 (11)	
H2A	0.9045	0.8496	0.4374	0.042*	
H2B	0.9083	0.6431	0.4413	0.042*	
C3	0.6645 (5)	0.9084 (8)	0.4485 (3)	0.0458 (14)	
H3A	0.6851	1.0154	0.4748	0.055*	
H3B	0.5905	0.9126	0.4387	0.055*	
C4	0.7193 (4)	0.8955 (6)	0.3750 (2)	0.0283 (10)	
H4A	0.7610	0.9999	0.3681	0.034*	
H4B	0.6690	0.8887	0.3333	0.034*	
C5	0.6608 (5)	0.5869 (8)	0.4562 (3)	0.0491 (15)	
H5A	0.5875	0.5894	0.4434	0.059*	
H5B	0.6748	0.4858	0.4885	0.059*	
C6	0.7217 (4)	0.5720 (6)	0.3848 (3)	0.0323 (11)	
H6A	0.7654	0.4684	0.3877	0.039*	
H6B	0.6745	0.5591	0.3415	0.039*	
C7	0.8408 (4)	0.7146 (7)	0.3008 (3)	0.0378 (12)	
H7A	0.8807	0.6065	0.3019	0.045*	
H7B	0.7888	0.7047	0.2603	0.045*	
C8	0.9111 (5)	0.8654 (8)	0.2840 (3)	0.0492 (14)	
H8A	0.9638	0.8778	0.3238	0.059*	
H8B	0.8722	0.9743	0.2803	0.059*	
N1	0.6916 (4)	0.7512 (5)	0.4966 (2)	0.0344 (10)	
H1C	0.6570	0.7580	0.5404	0.041*	
N2	0.7874 (3)	0.7339 (5)	0.3752 (2)	0.0262 (8)	
O1	0.4055 (3)	0.7844 (4)	0.65616 (19)	0.0342 (8)	
H1D	0.3713	0.7503	0.6914	0.051*	
O2	0.5835 (3)	0.8397 (4)	0.6163 (2)	0.0407 (9)	
O3	0.5392 (3)	0.5469 (4)	0.6708 (2)	0.0401 (9)	
O4	0.5490 (3)	0.8038 (5)	0.7549 (2)	0.0438 (9)	
H4C	0.5100	0.8835	0.7667	0.066*	
O5	0.2784 (3)	0.9370 (5)	0.8525 (3)	0.0589 (11)	
H5C	0.3371	0.9707	0.8448	0.088*	
O6	0.2824 (3)	0.6928 (6)	0.7558 (2)	0.0578 (12)	
O7	0.3544 (4)	0.6584 (6)	0.8901 (2)	0.0622 (12)	
H7C	0.3639	0.5548	0.8794	0.093*	
O8	0.1613 (4)	0.6852 (7)	0.8600 (2)	0.0734 (15)	
H8D	0.1511	0.7297	0.9099	0.110*	
Br1	0.11078 (4)	0.73719 (7)	0.02823 (3)	0.03808 (18)	
Br2	0.97424 (5)	0.81384 (9)	0.18848 (3)	0.0491 (2)	
P1	0.52097 (10)	0.74245 (15)	0.67295 (7)	0.0260 (3)	
P2	0.26999 (11)	0.73716 (17)	0.83560 (8)	0.0336 (3)	

### Atomic displacement parameters (Å^2^)

**Table:**

	*U*^11^	*U*^22^	*U*^33^	*U*^12^	*U*^13^	*U*^23^
C1	0.053 (4)	0.061 (4)	0.027 (3)	−0.003 (3)	−0.015 (2)	0.004 (2)
C2	0.027 (3)	0.037 (3)	0.041 (3)	−0.001 (2)	−0.010 (2)	−0.003 (2)
C3	0.054 (3)	0.045 (3)	0.039 (3)	0.018 (3)	0.011 (3)	0.008 (2)
C4	0.034 (3)	0.025 (2)	0.026 (2)	0.0043 (19)	−0.0025 (19)	0.0012 (18)
C5	0.064 (4)	0.044 (3)	0.040 (3)	−0.024 (3)	0.007 (3)	−0.005 (3)
C6	0.038 (3)	0.023 (2)	0.035 (3)	−0.004 (2)	−0.008 (2)	−0.0012 (19)
C7	0.040 (3)	0.038 (3)	0.035 (3)	0.004 (2)	0.004 (2)	−0.008 (2)
C8	0.048 (3)	0.060 (4)	0.040 (3)	−0.005 (3)	0.017 (3)	−0.013 (3)
N1	0.045 (3)	0.032 (2)	0.026 (2)	−0.0003 (18)	0.0029 (19)	0.0015 (16)
N2	0.027 (2)	0.0226 (18)	0.029 (2)	0.0039 (15)	−0.0020 (16)	−0.0014 (15)
O1	0.0329 (19)	0.0411 (19)	0.0284 (17)	0.0070 (15)	−0.0029 (15)	0.0076 (15)
O2	0.052 (2)	0.0301 (18)	0.042 (2)	−0.0086 (16)	0.0173 (17)	0.0011 (15)
O3	0.047 (2)	0.0254 (17)	0.048 (2)	0.0046 (15)	0.0133 (17)	0.0055 (15)
O4	0.041 (2)	0.058 (2)	0.0319 (19)	0.0148 (18)	−0.0095 (16)	−0.0110 (17)
O5	0.053 (2)	0.040 (2)	0.085 (3)	−0.0016 (19)	0.014 (2)	−0.010 (2)
O6	0.054 (3)	0.088 (3)	0.033 (2)	−0.016 (2)	0.0131 (19)	−0.014 (2)
O7	0.084 (3)	0.047 (2)	0.055 (3)	0.016 (2)	−0.007 (2)	−0.005 (2)
O8	0.063 (3)	0.113 (4)	0.045 (2)	−0.044 (3)	0.020 (2)	−0.022 (3)
Br1	0.0384 (3)	0.0382 (3)	0.0377 (3)	0.0004 (2)	0.0016 (2)	−0.0041 (2)
Br2	0.0453 (4)	0.0619 (4)	0.0406 (3)	−0.0004 (3)	0.0118 (3)	−0.0015 (3)
P1	0.0294 (6)	0.0237 (6)	0.0251 (6)	0.0022 (5)	0.0035 (5)	0.0006 (4)
P2	0.0386 (7)	0.0318 (7)	0.0308 (7)	−0.0040 (5)	0.0064 (6)	−0.0031 (5)

### Geometric parameters (Å, °)

**Table:**

C1—N1	1.488 (7)	C7—C8	1.500 (8)
C1—C2	1.526 (8)	C7—N2	1.517 (6)
C1—H1A	0.9700	C7—H7A	0.9700
C1—H1B	0.9700	C7—H7B	0.9700
C2—N2	1.501 (6)	C8—Br2	1.945 (5)
C2—H2A	0.9700	C8—H8A	0.9700
C2—H2B	0.9700	C8—H8B	0.9700
C3—N1	1.501 (6)	N1—H1C	0.9100
C3—C4	1.510 (7)	O1—P1	1.549 (4)
C3—H3A	0.9700	O1—H1D	0.8200
C3—H3B	0.9700	O2—P1	1.507 (3)
C4—N2	1.511 (6)	O3—P1	1.505 (3)
C4—H4A	0.9700	O4—P1	1.556 (4)
C4—H4B	0.9700	O4—H4C	0.8200
C5—N1	1.486 (6)	O5—P2	1.550 (4)
C5—C6	1.519 (7)	O5—H5C	0.8200
C5—H5A	0.9700	O6—P2	1.468 (4)
C5—H5B	0.9700	O7—P2	1.554 (4)
C6—N2	1.509 (6)	O7—H7C	0.8200
C6—H6A	0.9700	O8—P2	1.539 (4)
C6—H6B	0.9700	O8—H8D	0.9600
			
N1—C1—C2	109.1 (4)	C8—C7—H7B	108.9
N1—C1—H1A	109.9	N2—C7—H7B	108.9
C2—C1—H1A	109.9	H7A—C7—H7B	107.7
N1—C1—H1B	109.9	C7—C8—Br2	107.2 (4)
C2—C1—H1B	109.9	C7—C8—H8A	110.3
H1A—C1—H1B	108.3	Br2—C8—H8A	110.3
N2—C2—C1	109.9 (4)	C7—C8—H8B	110.3
N2—C2—H2A	109.7	Br2—C8—H8B	110.3
C1—C2—H2A	109.7	H8A—C8—H8B	108.5
N2—C2—H2B	109.7	C5—N1—C1	109.8 (4)
C1—C2—H2B	109.7	C5—N1—C3	110.0 (4)
H2A—C2—H2B	108.2	C1—N1—C3	110.0 (4)
N1—C3—C4	109.3 (4)	C5—N1—H1C	109.0
N1—C3—H3A	109.8	C1—N1—H1C	109.0
C4—C3—H3A	109.8	C3—N1—H1C	109.0
N1—C3—H3B	109.8	C2—N2—C6	108.0 (4)
C4—C3—H3B	109.8	C2—N2—C4	108.8 (3)
H3A—C3—H3B	108.3	C6—N2—C4	109.3 (4)
C3—C4—N2	110.1 (4)	C2—N2—C7	112.3 (4)
C3—C4—H4A	109.6	C6—N2—C7	107.2 (4)
N2—C4—H4A	109.6	C4—N2—C7	111.1 (4)
C3—C4—H4B	109.6	P1—O1—H1D	109.5
N2—C4—H4B	109.6	P1—O4—H4C	109.5
H4A—C4—H4B	108.2	P2—O5—H5C	109.5
N1—C5—C6	109.0 (4)	P2—O7—H7C	109.5
N1—C5—H5A	109.9	P2—O8—H8D	109.0
C6—C5—H5A	109.9	O3—P1—O2	112.23 (19)
N1—C5—H5B	109.9	O3—P1—O1	110.5 (2)
C6—C5—H5B	109.9	O2—P1—O1	108.1 (2)
H5A—C5—H5B	108.3	O3—P1—O4	106.7 (2)
N2—C6—C5	110.2 (4)	O2—P1—O4	111.2 (2)
N2—C6—H6A	109.6	O1—P1—O4	108.1 (2)
C5—C6—H6A	109.6	O6—P2—O8	110.1 (3)
N2—C6—H6B	109.6	O6—P2—O5	113.7 (3)
C5—C6—H6B	109.6	O8—P2—O5	104.8 (3)
H6A—C6—H6B	108.1	O6—P2—O7	114.4 (3)
C8—C7—N2	113.5 (4)	O8—P2—O7	111.0 (3)
C8—C7—H7A	108.9	O5—P2—O7	102.3 (2)
N2—C7—H7A	108.9		
			
N1—C1—C2—N2	−3.1 (6)	C1—C2—N2—C4	61.1 (5)
N1—C3—C4—N2	−2.8 (6)	C1—C2—N2—C7	−175.5 (4)
N1—C5—C6—N2	−4.0 (6)	C5—C6—N2—C2	62.0 (5)
N2—C7—C8—Br2	178.6 (3)	C5—C6—N2—C4	−56.2 (5)
C6—C5—N1—C1	−58.3 (6)	C5—C6—N2—C7	−176.8 (4)
C6—C5—N1—C3	62.8 (6)	C3—C4—N2—C2	−57.6 (5)
C2—C1—N1—C5	62.7 (5)	C3—C4—N2—C6	60.1 (5)
C2—C1—N1—C3	−58.4 (5)	C3—C4—N2—C7	178.2 (4)
C4—C3—N1—C5	−59.0 (6)	C8—C7—N2—C2	−60.4 (6)
C4—C3—N1—C1	62.0 (6)	C8—C7—N2—C6	−178.9 (4)
C1—C2—N2—C6	−57.5 (5)	C8—C7—N2—C4	61.8 (6)

### Hydrogen-bond geometry (Å, °)

**Table:**

*D*—H···*A*	*D*—H	H···*A*	*D*···*A*	*D*—H···*A*
N1—H1C···O2	0.91	1.79	2.667 (6)	162
O1—H1D···O6	0.82	1.71	2.519 (5)	170
O4—H4C···O3^i^	0.82	1.80	2.560 (5)	155
O5—H5C···O3^i^	0.82	1.73	2.555 (5)	179
O7—H7C···O2^ii^	0.82	1.77	2.555 (5)	159
O8—H8D···Br1^iii^	0.96	2.18	3.100 (4)	160

Symmetry codes: (i) −*x*+1, *y*+1/2, −*z*+3/2; (ii) −*x*+1, *y*−1/2, −*z*+3/2; (iii) *x*, *y*, *z*+1.

## References

[bb1] Chen, L.-Z., Zhao, H., Ge, J.-Z., Wu, D.-H. & Xiong, R.-G. (2008). *Cryst. Growth Des.* **9**, 3828–3831.

[bb2] Fu, D.-W., Ge, J.-Z., Dai, J., Ye, H.-Y. & Qu, Z.-R. (2009). *Inorg. Chem. Commun.* **12**, 994–997.

[bb3] Katrusiak, A. & Szafranski, M. (1999). *Phys. Rev. Lett.* **82**, 576–579.

[bb4] Rigaku (2005). *CrystalClear* Rigaku Corporation, Tokyo, Japan.

[bb5] Sheldrick, G. M. (2008). *Acta Cryst.* A**64**, 112–122. 10.1107/S0108767307043930 18156677

[bb6] Szafranski, M. & Katrusiak, A. (2008). *J. Phys. Chem. B*, **112**, 6779–6785. 10.1021/jp801106m 18465896

[bb7] Zhao, H., Qu, Z.-R., Ye, H.-Y. & Xiong, R.-G. (2008). *Chem. Soc. Rev.* **37**, 84–100. 10.1039/b616738c 18197335

